# An Update on Circular RNA in Pediatric Cancers

**DOI:** 10.3390/biomedicines11010036

**Published:** 2022-12-23

**Authors:** Angela Galardi, Marta Colletti, Alessandro Palma, Angela Di Giannatale

**Affiliations:** 1 Department of Pediatric Hemato-Oncology and Cell and Gene Therapy, IRCCS, Bambino Gesù Children’s Hospital, Viale San Paolo 15, 00146 Rome, Italy; 2 Translational Cytogenomics Research Unit, IRCCS, Bambino Gesù Children’s Hospital, Viale San Paolo 15, 00146 Rome, Italy

**Keywords:** circRNA, pediatric cancer, biomarker

## Abstract

Circular RNAs (circRNAs) are a class of single-stranded closed noncoding RNA molecules which are formed as a result of reverse splicing of mRNAs. Despite their relative abundance, only recently there appeared an increased interest in the understanding of their regulatory importance. Among their most relevant characteristics are high stability, abundance and evolutionary conservation among species. CircRNAs are implicated in several cellular functions, ranging from miRNA and protein sponges to transcriptional modulation and splicing. Additionally, circRNAs’ aberrant expression in pathological conditions is bringing to light their possible use as diagnostic and prognostic biomarkers. Their use as indicator molecules of pathological changes is also supported by their peculiar covalent closed cyclic structure which bestows resistance to RNases. Their regulatory role in cancer pathogenesis and metastasis is supported by studies involving human tumors that have investigated different expression profiles of these molecules. As endogenous competitive RNA, circRNAs can regulate tumor proliferation and invasion and they arouse great consideration as potential therapeutic biomarkers and targets for cancer. In this review, we describe the most recent findings on circRNAs in the most common pediatric solid cancers (such as brain tumors, neuroblastomas, and sarcomas) and in more rare ones (such as Wilms tumors, hepatoblastomas, and retinoblastomas).

## 1. Introduction

Circular RNAs (circRNAs) are single-stranded covalently closed RNAs that have been identified in several species such as viruses, prokaryotes, unicellular eucaryotes, and mammals [1,2,3,4]. Thanks to the advancements in sequencing techniques and the bioinformatics approach, it has been recognized that circRNAs are highly enriched in the human transcriptome and may play different functions. Indeed, they can act as protein scaffolds or miRNA sponges and can be translated in polypeptides. These molecules are characterized by high structural stability and are mainly localized in the cytoplasm of several cell types [5], with tissue- and developmental stage-specific expression [6]. Furthermore, several reports showed that circRNAs may play a key role in different pathological conditions, including neurological disorders [7], diabetes mellitus, cardiovascular diseases, inflammatory diseases, and tumors [8,9].

In particular, emerging body of evidence has shown that abnormal expression of circRNAs is tightly related to tumorigenesis [10]. In this context, the elucidation of circRNAs’ functional role and the identification of pathways they regulate in cancer can be fundamental for the discovery of new biomarkers and for the development of new targeted therapies. In this review, we reported the most recent findings on the role of circRNAs in solid pediatric tumors (Summarized in Table 1).

### Biogenesis, Regulation, and Functions of CircRNAs

**Figure 1:**
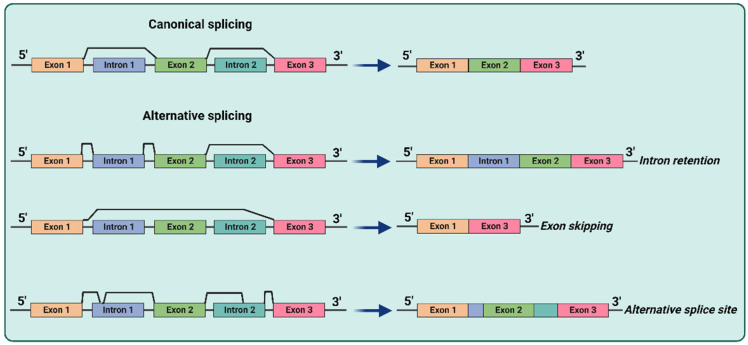
Schematic diagram depicting canonical splicing and the various types of alternative splicing (intron retention, exon skipping, and exon rearrangement following the use of alternative 3′ and 5′ splice sites).

Splicing is the process by which introns are removed from pre-mRNA to connect exons together to produce mature mRNA for the expression of most eukaryotic genes. The different combination of exons in mRNA producing diversified mature mRNA is called alternative splicing, which is a highly regulated mechanism depending on many trans-acting factors and cis-acting elements. RNA splicing occurs within the nucleus and is operated by an enzyme protein complex called spliceosome which recognizes the junction of introns and exons. The spliceosome consists of several small nuclear ribonucleoprotein particles (snRNPs) and numerous protein factors which recognize specific sequences at the 5′ and 3′ splice sites. The specific association of snRNPs to certain splice sites at the junctions of exons and introns initiates a series of reactions which lead to the removal of the intron and the union of two exons between which the intron was located to form linear mRNA (canonical splicing, Figure 1). Alternative splicing is a ubiquitous regulatory mechanism of gene expression that allows generation of more than one unique mRNA species from a single gene. Alternative splicing can generate mRNAs that differ in untranslated regions (UTR) or the coding sequence, with mechanisms including intron retention, exon skipping (removal of specific exons, referred to as cassette exons, that can be either included or excluded from the mature transcript depending on alternative splicing decisions), or the usage of alternative splice sites (that affects the boundaries between the introns and exons involved in this alternative splicing event contributing to transcript diversity) and intron retention (alternative splicing, Figure 1).

Circular RNAs (circRNAs) include a large class of noncoding RNAs derived from a noncanonical splicing event named back splicing where a downstream splice donor site is covalently linked to an upstream splice acceptor site [8]. CircRNAs are composed of 1–5 exons and the introns flanking the exons are up to three times as long as their linear counterparts [11]. Several studies emphasized the existence of many complementary inverter Alu repeats in intron segments, suggesting that such conditions facilitate and promote circularization [12]. Alu elements, generally located in introns, are abundant and constitute 11% of the reference human genome [13], and a recent analysis has indicated that repetitive elements, such as Alu repeats flanking exons, are responsible for RNA circularization and circRNA formation [14]. In fact, it has been reported that pairs of Alu repeats are enriched in the introns flanking exons that produce circRNAs [12,15], which is further confirmed by the fact that the majority of circRNA sequence is complementary to Alu sequences [16]. This was also proved in vitro by mutagenized plasmids that express different human circular RNAs, where the authors found that splicing sites and short inverted repeated sequences were sufficient to circularize RNA and that mutating some nucleotides inside the Alu elements also prevented circularization [17]. Zhang et al. also confirmed that the complementarity and pairing between Alu elements with reverse orientation regulates the expression of circular RNAs [16,18]. In particular, the adenosine-to-inosine editing is facilitated by double-stranded Alu sequences, thus promoting RNA circularization [16,19].

**Figure 2:**
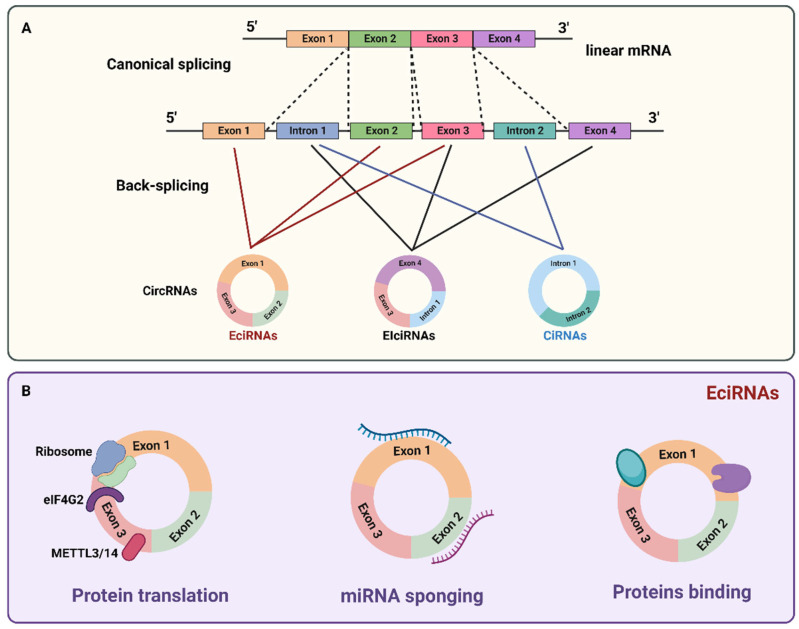
(**A**) Biogenesis of circRNAs: circRNAs are created by noncanonical splicing process known as back splicing. A downstream splice donor is joined to an upstream splice acceptor. Exonic circRNAs (EciRNAs) are formed via back splicing (red lines) with multiple exons or a single exon, the major forms of circRNAs. Exon–intron circRNAs (EIciRNAs) are circularized with the retained intronic sequences between the circularized exons, predominantly in the nucleus (black lines). Intronic circRNAs (ciRNAs) are formed from intronic lariat precursors escaping from the debranching step of canonical linear splicing, abundant in the nucleus (blu lines). (**B**) CircRNA are involved in gene expression regulation through distinct mechanisms: the processing of circRNAs affects splicing of their linear mRNA counterparts, can regulate transcription and translation of their parental genes. The association of translation initiation factor eIF4G2 with circRNA triggers its translation, and this process can be enhanced by METTL3/14. Furthermore, circRNAs can act as sponges for miRNAs through their binding sites or interact with different proteins to form specific circRNPs that subsequently influence modes of action of the associated proteins.

CircRNAs are subdivided into three classes depending on their synthesis: exonic (EciRNAs), intronic (CiRNAs), and exon–intron circRNAs (EIciRNAs) [20]. The biogenesis of circRNAs is closely linked to the alignment and the scrambling of exon and intron parties [21]. EciRNAs are synthesized via back splicing of the 5′ splice site to a 3′ splice site. CiRNAs are formed from intronic lariat precursors escaping from the debranching step of canonical linear splicing while CiRNAs synthesis is different as it depends on GU-rich sequences near the 5′ splice site and C-rich sequences near the branch point. To form mature circRNA, during the back splicing event, first, the two segments bind to form a circular structure followed by the incision of exonic and intronic sequences in the binding portion of the spliceosome, and after that, the residual introns are stitched together [22]. CircRNA biogenesis relies on canonical splicing machinery, and their expression level can be regulated in cells at three levels: precursor RNA transcription, post- or co-transcriptional processing, and turnover. If canonical splicing happens first, it generates a linear RNA with skipped exons and a long intron lariat containing these skipped exons, which are then back-spliced to generate circRNA (exon skipping or lariat intermediate model). If back splicing happens first, it directly generates circRNA together with an exon–intron(s)–exon intermediate that can meet two fates: be further processed to produce linear RNA with skipped exons or be degraded (direct back splicing model). In both models, the expression of circRNA is associated with an alternatively spliced linear RNA with exon exclusion, and the way in which the spliceosome chooses either canonical splicing or back splicing to start the production of circRNA is still unclear (Figure 2A). These two steps may happen stochastically or synergistically. To bring the downstream donor and upstream acceptor sites close together to promote back splicing, both cis-elements and trans-factors are required because back splicing is unfavorable for spliceosome assembly and is less efficiently catalyzed. The majority of circRNAs are formed from one or more exons of known protein-coding genes. Products resulting from all of the basic types of alternative splicing of linear RNA can be found within circRNAs, and some circRNAs contain exons that are not included in linear transcripts. Despite the lack of polyadenylation (poly(A)) and capping, circRNAs generally localize to the cytoplasm. However, internal intron retention may lead to the production of circRNAs that contain sequences derived from both exons and introns [23]. Although they are expressed at low levels, recent discoveries have revealed a wide range of regulatory functions performed by these circular molecules. CircRNA are involved in gene expression regulation through distinct mechanisms: the processing of circRNAs affects splicing of their linear mRNA counterparts, can regulate transcription of their parental genes or splicing of their linear cognate. Some circRNAs containing the internal ribosome entry site (IRES) can be translated into proteins through a process that involves the N^6^-methyladenosine (m^6^A) RNA base modification, eukaryotic translation initiation factor 4 gamma (eIF4G2), methyltransferase-like 3 (METTL3)/methyltransferase-like 14 (METTL14), and the association of the ribosome. Furthermore, circRNAs can act as endogenous competitive RNAs (ceRNAs) defined as miRNA sponges: they bind to the corresponding miRNAs via their complementary sequences, thereby regulating downstream target gene expression which is inhibited by miRNAs. CircRNAs can also interact with different proteins to form specific circRNPs that subsequently influence modes of action of the associated proteins (Figure 2B).

## 2. CircRNAs in Cancer

CircRNAs have been found to be dysregulated in cancer and involved as oncogenes or tumor suppressors in tumor development and progression. Since they have a different expression in tumors compared to normal tissues [21,22,23], they represent potential diagnostic/prognostic biomarkers and interesting targets for the development of new therapeutic approaches. CircRNAs may affect tumor progression acting in different ways: as miRNA sponges by sequestering specific miRNAs to regulate their downstream targets, thereby suppressing or promoting tumor progression [24,25], or interacting directly with specific proteins to regulate their or their targets’ function/translation. This mechanism allows the activation or inhibition of downstream pathways to affect malignant behavior of tumors [26,27]. Furthermore, it has been suggested that circRNA could be selectively translated from the same linear RNA, though only few circRNA translation products have been actually detected [28,29].

### 2.1. Pediatric Brain Tumors

#### 2.1.1. Medulloblastoma

Medulloblastoma (MB) is one of the most common malignant tumors affecting the central nervous system (CNS) in children. Although considerable progress has been achieved with the improvement of classic therapeutic approaches such as surgery, radiotherapy, and chemotherapy, the prognosis of some patients with MB remains very poor. Therefore, highly specific molecular targeted treatment, which can improve therapeutic efficacy and reduce side effects in patients, has become a research hotspot. Among the molecules with a potential prognostic/therapeutic value, circRNAs are also emerging in MB. The first evidence of circRNA involvement in the regulation of proliferation and growth of MB was reported in 2018 by Lv et al. With a next-generation sequencing approach on RNAs extracted from four human MBs and four human normal cerebellum tissues, 44,184 distinct circRNAs were identified, and among those, two were expressed differently between the two groups: circ-SKA3 and circ-DTL. Silencing studies on DAOY cells using si-circ-SKA3 and si-circ-DTL plasmids demonstrated that the downregulation of expression of these two circRNAs suppressed cell proliferation, migration, and invasion in human MB cell lines [30]. In order to deepen the role of circSKA3 in MB, Wang et al. confirmed a higher level of these molecules in MB tissues compared to the adjacent normal tissues and demonstrated the relationship between circSKA3 and its predicted target miR-383-5p, whose expression is reduced in MB tissues. In particular, the authors showed that circSKA3 facilitated MB progression through the suppression of miR-383-5p by acting as a sponge for this miRNA. This action leads to the regulation of FOXM1 expression and accelerates MB progression. In conclusion, these data suggest that circSKA3 depletion blocks MB progression by regulating FOXM1 expression via miR-383-5p [31]. Last year, it was demonstrated that circ-SKA3 decoyed miR-326 to increase the ID3 expression leading to cell proliferation, migration, and invasion in MB [32]. Moreover, it was observed that circSKA3 expression in MB tissues was negatively correlated with the miR-520 expression and positively correlated with the CDK6 expression, suggesting that circSKA3 facilitates MB progression through the miR-520 h/CDK6 axis [33].

#### 2.1.2. Ependymoma

Ependymomas represent one of the most common CNS tumors among children, even though they are rare. This malignancy occurs predominantly intracranially (supratentorial brain and posterior fossa) in children between 0 and 4 years of age, but it can also arise in older children and adults. Patients with intracranial ependymomas have high morbidity and mortality rates, and clinical management is challenging. A profiling of circRNAs was performed in a cohort of 10 pediatric patients diagnosed with ependymoma and three healthy controls in 2018. The authors found a significant downregulation of circRNAs in the ependymoma tissues compared to the healthy control samples, with 1167 circRNAs that showed a high abundance, of which 263 were detected in the ependymomas and 1126—in the controls. Following bioinformatic analysis, five circRNAs were found to be upregulated: circRMST, circLRBA circWDR78, circDRC1, and circBBS9 [34].

### 2.2. Neuroblastoma

Neuroblastoma (NB) is the most common extracranial solid cancer in childhood originating from primitive neuronal crest cells of the sympathetic nervous system and typically develops in the adrenal medulla or the paraspinal ganglia. NB is a complex tumor capable of rapid progression or spontaneous resolution, the latter usually occurring in under-one children. In 25% of the cases, a solitary mass that may be cured by surgery is present, whereas about 60% of the patients present disseminated disease at diagnosis, involving mainly bone, bone marrow, lymph nodes, and liver. In patients with metastasis, survival is poor, with a high mortality rate [35]. Despite scientific advances contributing to an increased understanding of genetic, molecular, and cellular mechanisms in NB, the prognosis of children with regional or distant metastasis disease remains still poor. Thus, efforts are being made to unveil the mechanisms underlying NB progression in order to identify new therapeutic targets. Even though in the past decade some evidence showed that noncoding RNAs have a role in the regulation of different cellular processes such as chromatin remodelling, transcription, transduction, and post-transcriptional modification, the role of circular RNAs in NB is just starting to emerge. In 2019, Chen et al. identified one circRNA, circAGO2 (has_circ_0135889), that was upregulated in several cancer tissues, including NB. In particular, they observed that the overexpression of circAGO2 is associated with the promotion of growth and invasion of cancer cell lines in vitro and poor prognosis of patients. The promotion of growth and invasiveness are related to the interaction between circAGO2 and the human antigen R (HuR) protein which implies the competitive enrichment on the 3′ untranslated region (UTR) of target genes, resulting in the reduction of AGO2 binding, repression of AGO2/miRNA-mediated gene silencing, and promotion of tumorigenesis and aggressiveness [36]. Shortly after this observation, CUX1-generated intron-containing circular RNA (circ-CUX1) was identified by Li et al. as a circRNA with oncogenic properties in NB cell lines. In particular, elevated circ-CUX1 promotes aerobic glycolysis, growth, and aggressiveness of NB cells by binding to EWS RNA-binding protein 1 (EWSR1) and facilitating its interaction with the MYC-associated zinc finger protein (MAZ); this results in transactivation and transcriptional alteration of CUX1 [37]. CUX1 is a transcriptional factor regulating glycolytic genes such as ENO1, GPI, and PGK1 in NB, and its elevated expression has been reported to be associated with poor prognosis in several tumors [38]. Zhang et al. observed that miR-16-5p is downregulated in NB cells and tissues, and it is a direct target of circ-CUX1. Thus, circ-CUX1 acts as a sponge for miR-16-5p, which, in turn, no longer inhibits the messenger of DMRT2, which is upregulated, accelerating proliferation, migration, invasion, and glucose uptake in NB cells [39]. The target prediction approach revealed miR-388-3p as another possible target for circCUX1. In 2021, Wang et al. confirmed the role of circCUX1 in triggering the progression and glycolysis of NB by serving as competing endogenous RNA (ceRNA) for miR-388-3p and upregulating plant homeodomain finger protein 20 (PHF20) [40]. Another circRNA upregulated in NB tissues and associated with a poor prognosis is circDGKB (has_circ_0133622). Overexpression of this molecule promotes proliferation, migration, and invasion of SK-N-SH cells, inhibits apoptosis, and induces S phase arrest, while its downregulation reverts these effects. In particular, circDGKB targets miR-873, which is an oncosuppressor in several cancers. The loss of the inhibitory effect exerted by miR-873 on its target GLI1 (glioma-associated oncogene 1) leads to increased proliferation, migration, and invasion in NB cells [41]. Zhang et al. collected RNA sequencing data of 39 NB cell lines and two normal cell lines (RPE-1 and fetal brain) as the control and identified 39,022 functional circRNAs [42]. From their analysis, it emerged that circRNAs located within the hotspot at 2p originated from the genes adjacent to MYCN, probably deriving from *MYCN* amplification. In particular, they found 29 circRNAs dysregulated in NB; those located within the amplified regions of MYCN were upregulated in the cell lines with MYCN amplification and a high activation level of MYCN targets. Furthermore, they identified has_circ_0002343, which is implicated in the regulation of PI3K/Akt/mTOR signaling, and has_cir_0001361 was associated with the genes implicated in epithelial-to-mesenchymal transition (EMT), such as NOTCH2, SERPINHI, LAMCI [43]. In 2021, Lin et al. performed high-throughput RNA sequencing of five paired NB tumors and adjacent normal adrenal medulla samples and identified 4704 differentially expressed circRNAs (2462 up- and 2242 downregulated). Among the differentially expressed circRNAs, 10 highly downregulated circRNAs were confirmed to be less expressed in 20 NB specimens compared to the adjacent normal adrenal gland samples. Moreover, the authors chose to extend the analysis to seven other circRNAs that target miR-21, which plays an important role in the regulation of proliferation and apoptosis in NB [44]. Three miR-21-related circRNAs (circRNA-TBC1D4, circRNA-NAALAD2, circRNA-TGFBR3) were significantly downregulated in NB samples. In particular, circRNA-TBC1D4 was associated with the MYCN status, circRNA-TGFBR3—with the histological classification. In order to investigate the biological functions of these circRNAs, the authors overexpressed circRNA-TBC1D4 in the SH-SY5Y NB cell lines and observed a significant decrease in the migratory properties of the cells [45]. Two papers published in 2021 illustrated that nuclear protein 4 like (NOL4L) was regulated by microRNAs, which were regulated, in turn, by circular RNAs. In the first work, miR-362-5p, which has suppressive functions in NB progression, was sponged by circRNA phosphodiesterase 5 A (circPDE5A, has_circ_0002474), which, in turn, was upregulated in NB cells and tissues. Among the functional targets of miR-362-5p, the authors identified NOL4L, whose expression correlates with the circPDE5A levels in NB [46]. In the second work, NOL4L was negatively regulated by miR-432-5p and positively regulated by circ_0132817. Inhibition of circ_0132817 restrained tumor growth by upregulating miR-432-5p and downregulating NOL4L, suggesting that circ_0132817 knockdown inhibits the progression of NB through the modulation of the miR-432-5p/NOL4L axis [47]. In 2022, Tang et al. performed a high-throughput microarray for circRNAs on three NB and three gangliocytoma tissues [48]. From their analysis, circ0125803 emerged as the most upregulated circRNA. Circ0125803 interacts with miR-197-5p, which has E2F1 as the target, and the upregulation of this gene promotes NB progression. Yang et al. observed that in NB tumor specimens and cells, circ_0135889 and neuronal differentiation 1 (NEUROD1) were upregulated while miR-127-5p was down-regulated. Cell proliferation, migration, and invasion were suppressed following circ_0135889 silencing. Circ_0135889 acts as a sponge for miR-127-5p, and inhibition of miR-127-5p counteracts the inhibitory impact of circ_0135889 knockdown on the malignant behavior of NB cells. Moreover, NEUROD1 was a direct target of miR-127-5p, and miR-127-5p exerted antitumor activity in NB cells by targeting NEUROD1 [49]. The expression level of circular RNA (circRNA) kinesin superfamily protein 2A (circKIF2A, also known as hsa_circ_0129276) has been reported to be upregulated in NB tissues, SK-N-AS and LAN-6 cell lines. Silencing of circKIF2A inhibits cell proliferation, migration, invasion, and glycolysis, boosts apoptosis in NB cells in vitro, and blocks the growth in vivo. CircKI2A acts as a sponge of miR-377-3p, targeting phosphoribosyl pyrophosphate synthetase 1 (PRPS1), leading to an increase in its expression that determines a decreased aggressivity of NB cells [50]. Karami Fath et al. recently published a review where they reported the potential role of circRNA in NB progression [51]. This body of evidence underlines the potential of these molecules as possible biomarkers and therapeutic targets in NB, even though extensive validation is needed, in particular in relation to the individual characteristics of patients.

### 2.3. Sarcomas

#### 2.3.1. Rhabdomyosarcoma

Rhabdomyosarcoma (RMS) is the most-common soft tissue malignancy in children and adolescents, accounting for up to 3–4% of childhood cancer cases and approximately 50% of all sarcomas [52,53]. Among the main RMS subtypes, embryonal (ERMS) and alveolar sarcomas (ARMS) account for 60% and 20% of all RMS cases, respectively. ARMS is associated to specific genetic alterations and generally has a worse prognosis due to its low response to treatment [54]. In 2019, Rossi et al. reported that circ-ZNF609 is expressed in growing myoblasts, showing that its depletion leads to cell proliferation modulation. Indeed, circ-ZNF609 is upregulated in RMS cell lines, in particular in ARMS, and has a role as a positive regulator of cell proliferation pathways. Knockdown of circ-ZNF609 induces the block of the G1–S transition, specifically in ERMS cells, with a decrease in the p-AKT protein levels in both ERMS and ARMS cell types. Moreover, circ-ZNF609 has been described to be overexpressed in RMS primary tissues [55]. In 2021, the same research group described a high expression of circVAMP3 in ARMS cells lines and demonstrated its involvement in cell cycle progression through the alteration of AKT-related pathways. In particular, downregulation of circVAMP3 leads to the upregulation of CDKN1A and WEE1, which directly regulate the CCNB1/CDK1 complex, controlling the G2/M checkpoint, as well as the downregulation of AKT and ERK1, therefore leading to an accumulation of cells in the G2 phase [56].

#### 2.3.2. Osteosarcoma

Osteosarcoma (OS) accounts for approximately 35% of primary malignant bone tumors; its metastatic form presents the lowest survival rate out of all pediatric cancers despite the use of multiple chemotherapeutic regimens [57]. The MNAT1 protein (menage a trois 1, MAT1) is upregulated in different sarcomas (Ewing’s sarcoma, synovial sarcoma), but in particular in OS tissues compared to normal bone tissue. Among the four miRNAs (miRNA-26a-5p, miRNA-26b-5p, miRNA-200a-3p, miRNA-141-3p) that are aberrantly expressed in OS and are predicted to target MNAT1, miRNA-26a-5p expression shows negative correlation with MNAT1 expression. This effect is due to the action of has_circ-0001146 that promotes MNAT1 expression by sponging miR-26a-5p and promoting proliferation and invasion ability [58]. Zhang et al. reported that in 10 pediatric OS tissue samples, has_circ-0005909 was upregulated in respect to the adjacent normal tissue. This circRNA was confirmed to also be upregulated in OS cell lines MG63, HOS, 143B, and U2OS. Knockdown of circ_0005909 in OS cells demonstrated that this circRNA represses proliferation by targeting miR-338-3p that could bring the 3′UTR of HMGA1 (high mobility group A1) [59].

### 2.4. Wilms’ Tumor

Wilms’ tumor (WT, nephroblastoma) develops before the age of 3 years and accounts for 6% of childhood tumors and 95% of pediatric kidney tumors. In recent years, with the improvement of therapeutic strategy, around 85% of the patients affected by this tumor are cured, with a few children presenting relapse, metastasis, and chemoresistance who have a poor prognosis. Thus, the identification of new molecules such as circRNAs would be fundamental to improving the survival of patients with high-risk disease. In 2021, Cao et al. conducted high-throughput microarray sequencing to screen circRNAs in Wilms’ tumor cells and tissues [60]. Wilms’ tumor samples and the adjacent normal kidney samples were collected and analyzed for circRNA expression. Circ0093740 was identified as upregulated in the tumor samples in respect to the normal kidney samples and the Wilms’ tumor cell lines. Silencing of circ0093740 inhibited the proliferation and migration ability of the Wilms’ tumor cells, and this mechanism is mediated by the sponging of miR-136/145 operated by circ0093740 that leads to the upregulation of DNA (cytosine-5-)-methyltransferase 3 alpha (DNMT3A), transcript variant 3. An opposite effect was observed for has_circ_0008285 (circCDYL) which was downregulated in WT tissues compared to normal adjacent tissues, and its overexpression suppresses proliferation, migration, and invasion of WT cells in vitro and in vivo [61]. These effects are due to the upregulation of the tight junction protein 1 (TPJI) expression following the ability to circCDYL to sponge miR-145-5p. In 2022. Jiaju et al. observed that circSLC7A6 was upregulated in WT tumor samples and cells in respect to the controls. Cell apoptosis was increased while cell viability, migration, and invasion were repressed by circSLC7A6 silencing. Furthermore, miR-107 was a direct target of this circRNA, and circSLC7A6 could upregulate ABL2 expression by serving as a ceRNA of miR-107 [62].

### 2.5. Hepatoblastoma

Hepatoblastoma (HB), one of the major liver cancers in infants, accounts for about 1% of all pediatric cancers [63]. To date, surgical resection, adjuvant chemotherapy, and liver transplantation are the methods mainly used for HB treatment. However, a high percentage of patients have a high risk of relapse or metastasis, and the mortality rate in those advanced cases is still over 35% [64]. Furthermore, in the field of HB, considerable effort has been devoted to the identification of circRNAs. In 2018, Liu et al. compared the HB expression profile with the paired adjacent normal tissue samples and identified 869 differentially expressed circRNAs. Among these molecules, has_circ_0015756 was the most significantly upregulated both in tissues and in metastatic HB cell lines. A reduction of viability, proliferation, and invasion ability in SMMC-7221 and HepG2 HB cells was observed following the silencing of this circRNA. Predictive bioinformatic analyses suggested miR-6134, miR-7854-3p, miR-4778-3p, miR-1250-3p, and miR-4659a-3p as a putative miRNA target on circ_0015756. Functionality experiments suggested that miR-1250-3p directly regulates circ_0015756, and this was confirmed by the reduction of viability, proliferation, and invasion following miR-1250-3p mimic transfection in HuH-6 cells [65]. In 2019, two papers were published on dysregulated circRNAs in HB. Song et al. demonstrated by using qRT-PCR and in situ hybridization that has_circ_0000594 is upregulated in HB samples collected during surgery. This circRNA directly regulates miR-217, which is a well-recognized tumor suppressor miRNA, to act on SIRT1 (Sirtuin1) expression, this latter being an oncogene implicated in the promotion of the malignant phenotype [66]. Zhen et al. investigated by means of sequencing analyses the expression profile of circRNAs in HB tissues and discovered that circHMGCS1 (has_circ_0072391) is strongly upregulated in patients’ tissues and is negatively correlated with the prognosis. Furthermore, they found that this circRNA exerts its oncogenic role via sponging tumor suppressor miR-503-5p to upregulate the IGF–PI3K–Akt signaling and regulating glutamine metabolism [67]. Using circBase, Liu et al. looked at the circRNAs which are derived from the STAT3 gene sequence and that were expressed in HepG2 and HuH-6 cells compared to a normal liver cell line, THLE-3, and identified 14 sequences [68,69]. Among these sequences, circ_0043800 and circ_0043804 were highly expressed in cancer cells in respect to the control cell line and were also detected in HB and the adjacent nontumor tissues. Only circ_0043800 was significantly upregulated in cancer tissues, and the authors renamed it as circ-STAT3. The silencing of circ-STAT3 led to the inhibition of HB (HepG2 and HuH-6) cell growth and migration and a stem cell phenotype. Circ_0043800, that is predominantly located in the cytoplasm, was found to upregulate STAT-3 and Gli2 (GLI family zinc finger 2) via sponging miR-29a/b/c-3p, and this leads to the promotion of HB tumor growth [69]. In 2021, Chen et al. explored the role of circRNAs in stemness maintenance of HB cells. CircRNA CDR1 was highly expressed in HB cancer stem cells (CSC)-enriched population, and its knockdown decreased the proportion of stem cells. This effect was due to its sponge activity on miR-7-5p, which leads to the increase in Kruppel-like factor 4 (KLF-4) expression [70]. The expression of circSETD3 was investigated by Li et al. who observed a significant reduction in HB tissues and cell lines (HepG2 and HuH-6) compared to normal tissues and a liver cell line (THLE-3). Furthermore, low expression of circSETD3 has been shown in advanced stages of disease and to be associated with poor survival in HB patients. These results indicate that circSETD3 acts as a tumor suppressor in HB, and functional experiments on this tumor demonstrated that its expression inhibits cell proliferation, migration, epithelial-to-mesenchymal transition (EMT) and induces apoptosis via sponging miR-423-3p to promote Bim expression [71].

### 2.6. Retinoblastoma

Emerging evidence suggests that abnormal expression of circRNA is very closely related to the development and progression of various ocular disorders, including retinoblastoma (RB). RB, a malignancy originating from embryonal retinal cells, is the most common eye cancer occurring in the pediatric population. Several approaches have been employed to treat RB, with survival rates currently exceeding 95%. However, in the presence of resistant retinal, vitreous, or humor aqueous disease, the management of local tumor control and the visual outcome are still challenging. Therefore, the understanding of molecular events underlying the initiation and the progression of RB remains crucial for the treatment of this cancer. First evidence of the involvement of circRNAs in RB progression was provided in 2018 by Xing et al. with a work illustrating the clinical value and biological functions of has_circ_0001649. Has_circ_0001649 expression levels were significantly downregulated in RB cell lines in respect to retinal pigment epithelial (RPE) cell line ARPE-19 and in RB tissues compared with normal retinal tissues. Low expression of has_circ_0001649 was associated with an aggressive phenotype of RB and predicted a poor prognosis for RB patients after surgery [72]. Further functional studies demonstrated the ability of has_circ_0001649 to induce changes in proliferation and apoptosis in RB cells through the regulation of AKT/mTOR signaling. In 2019, Jiao et al. investigated the expression of circRNA in primary RB tissue samples compared to healthy retinas by means of RNA sequencing. They identified 557 differentially expressed circRNAs, with 550 circRNAs downregulated and seven circRNAs upregulated in RB tissues compared to the corresponding normal retinas. Gene Ontology (GO) enrichment analyses showed that the host genes of differentially expressed circRNAs were associated predominantly with chromatin modification and phosphorylation. In particular, among circRNAs whose host genes participated in chromatin modification, TET1-has_circ_0093996 was significantly downregulated in the RB samples. Prediction using TargetScat reveals that cluster miR-96-182-186 is sponged by TET1-has_circ_0093996 and that programmed cell death 4 (PDCD4) is a potential candidate gene impacted in RB tissue as the target of miR-183 [73]. In another work, Fu et al. observed that among the potential targets of circTET1, there are miR-492 and miR-494-3p in RB. The ability to sponge these miRNAs by circTET1 has as a consequence the inhibition of the Wnt/β-catenin signaling pathway and the inhibition of tumor progression [74]. In 2020, Wenbo et al. provided the evidence of the potential role of circ_0075804 in regulating RB cell proliferation by binding to heterogeneous nuclear ribonucleoprotein K (HNRNPK) and enhancing the stability of E2F3 mRNA [75]. Du Shanshan et al. identified that circ_ODC1 is implicated in the regulation of RB proliferation through a mechanism that involves miR-422a and S phase kinase-associated protein 2 (SKP2). In particular, they observed that circ_ODC1 sponging miR-422a, which targets SKP2 mRNA, indirectly increases the SKP2 protein to promote cell growth in RB [76]. Circ_0000527 has been demonstrated to be expressed at a high level in RB tissues and cells implicated in the promotion of RB progression. Specifically, circ_0000527, by sponging miR-646, increases the expression of its target BCL-2 through the promotion of malignant phenotypes on RB cells [77]. Among the messengers targeted by miR-646, there is also LDL receptor-related protein (LRP)-6 mRNA. Zhang et al. demonstrated that upregulation of miR-646 and knockdown of circ_0000527 induce a decrease in LRP6 expression in RB cells; on the other hand, inhibition of miR-646 or overexpression of circ_0000527 enhance LRP6 expression, promoting cell proliferation and migration [78]. Another mechanism by which circ_0000527 facilitates RB progression is through the regulation of the miR-98-5p/X-linked inhibitor of apoptosis (XIAP) axis. Indeed, the silencing of circ_0000527 suppresses proliferation, migration, and invasion of RB cells and promotes apoptosis by increasing expression of miR-98-5p that targets XIAP [79]. Recently, two papers have highlighted the role of circular RNA circ_0000034 in the development of RB. Liu et al. showed that the level of circ_0000034 and syntaxin (STX17) was increased in RB tissues and cells, while miR-361-3p was decreased, suggesting a role of the miR-361-3p/STX17 axis in promoting RB cell growth [80]. Sun et al. found that has_0000034 expression in RB tissues was high compared to normal retinal tissues and correlated with advanced disease in RB patients. Thus, they suggested that has_circ_0000034 may promote RB aggressiveness by sponging miR-361-3p [81]. Le et al. evaluated the expression level of circMKLN1 in 55 RB tissue samples, showing its downregulation compared to retinal tissue [82]. To evaluate the role of this circRNA in RB development, they transfected into Y79 and WERI-RB1 cells a vector overexpressed circMKLN1, showing inhibition of its target miR-425-5p and suppression of proliferation and aggressiveness of RB cells. This anticancer effect is due to the upregulation of oncosuppressor gene PDCD4 following the inhibition of miR-425-5p carried out by circMKLN1. Conversely, it was observed that circ-FAM158A was overexpressed in RB tissues and cells, and its knockdown inhibited RB cell growth, migration, and invasion. Moreover, in RB specimens and cells, miR-138-5p was lowly expressed, while solute carrier family 7 member 5 (SLC7A5) was highly expressed, suggesting a possible relationship between these two molecules. In fact, it was reported in RB that circ-FAM158A may act as an oncogene by sponging miR-138-5p to regulate *SLC7A5* expression [83]. In 2022, Guangwei analyzed the expression of four different circRNAs identified with bioinformatics analysis via the circBank database (circ_0084811, circ_0137212, circ_0137213, circ_0137214) in RB cell lines. Among the four, only circ_0084811 was expressed at a high level in HXO-Rb44, Y79, SO-Rb50, and WERI-Rb-1 in respect to the human retinal pigment epithelial ARPE-19 cell line. Functional assays demonstrated that circ_0084811 facilitated cell proliferation but inhibited cell apoptosis. Moreover, circ_0084811 regulated its host gene E2F transcription factor 5 (E2F5) whose expression at the protein and mRNA levels was reduced following circ_0084811 silencing. Furthermore, circ_ 0084811 deficiency induced cell apoptosis, and this effect could be partially countervailed by the knockdown of miR-18a-5p or miR-18b-5p while it could be greatly counteracted by E2F5 upregulation. These observations suggested that circ_0084811 regulates E2F5 expression via sponging miR-18a-5p and miR-18b-5p [84].

**Table 1:** CircRNAs involved in various pediatric cancers.

Tumor	CircRNA	Related Network	Function	Reference
MB	circ-DTL		Promotion of proliferation	[30]
		miR-383-5p	Promotion of proliferation	[30]
			Promotion of cancer progression	[31]
MB	circSKA3	miR-326	Increased proliferation	[32]
		miR-520 h	Promotion of cancer progression	[33]
EP	circRMST, circLRBA circWDR78, circDRC1, and circBBS9			[34]
NB	circAGO2 (hsa_circ_0135889)		Promotion of tumorigenesis and aggressiveness	[36]
NB	circCUX1	EWS RNA-binding protein	Promotion of glycolysis, growth, and aggressiveness	[37]
NB	circCUX1	miR-16-5p/DMTR2	Promotion of proliferation, migration, invasion, and glucose uptake	[39]
NB	circCUX1	miR-388-3p/PHF20	Promotion of cancer progression	[40]
NB	circDGKB (has_circ_0133622)	miR-873/GLI1	Increased proliferation, migration, and invasion	[41]
NB	has_circ_0002343	PI3K/Akt/mTOR signaling	Survival	[42]
NB	has_circ_0001361	NOTCH2, SERPINH1, LAMC1	Epithelial-to-mesenchymal transition	[44]
NB	circTBC1D4, circNAALAD2, circTGFBR3	miR-21	Decreased migratory properties	[45]
NB	circPDE5A (has_circ_0002474)	miR-362-5p/NOL4L	Promotion of proliferation and migration	[46]
NB	circ_0132817	miR-432-5p/NOL4L	Promotion of tumor progression	[45]
NB	circ0125803	miR-197-5p/E2F1	Promotion of tumor progression	[48]
NB	circ_0135889	miR-127-5p/NEUROD1	Promotion of proliferation and tumorigenicity	[49]
NB	circKIF2A (hsa_circ_0129276)	miR-377-3p/PRPS1	Promotion of cell proliferation, migration, invasion, and glycolysis	[50]
ERMS	circZNF609	AKT	Regulation of cell proliferation	[55]
ARMS	circVAMP3	AKT	Regulation of cell proliferation	[56]
OS	has_circ_0001146	miR-26a-5p/MNAT1	Promotion of proliferation and invasiveness	[58]
OS	has_circ_0005909	miR-338-3p/HMGA1	Promotion of cancer development	[59]
WT	circ0093740	miR-136/145/DNMT3A	Promotion of proliferation and migration ability	[60]
WT	circCDYL	miR-145-5p/TJP1	Reduction of cell proliferation, migration, and invasion	[61]
WT	circSLC7A6	miR-107/ABL2	Cancer promotion	[62]
HB	circ_0015756	miR-1250-3p	Increased viability, proliferation, and invasion	[63]
HB	has_circ_0000594	miR-217/SIRT1	Promotion of proliferation, viability, and migration	[66]
HB	circHMGCS1 (has_circ_0072391)	miR-503-5p/IGF-PI3K-Akt signaling	Regulation of glutamine metabolism	[67]
HB	circ_0043800	miR-29a/b/c-3p/STAT3/GLI1	Promotion of tumor growth	[69]
HB	CDR1as	miR-7-5p/KLF4	Promotion of proliferation and stemness	[70]
HB	circSETD3	miR-423-3p/Bim	Inhibition of proliferation, migration, and EMT and induction of apoptosis	[71]
RB	has_circ_0001649	AKT/mTOR	Regulation of proliferation and apoptosis	[72]
RB	TET1-has_circ_0093996	miR-183/PDCD4 miR-494/miR-494-3p	Chromatin modification Inhibition of tumor progression	[73] [74]
RB	circ_0075804	HNRNPK	Regulation of proliferation	[75]
RB	circ_ODC1	miR-422a/SKP2	Regulation of cell growth	[76]
		miR-646/BCL-2	Promotion of RB progression	[77]
RB	circ_0000527	miR-646/LRP6	Promotion of cell proliferation and migration	[78]
		miR-98-5p/XIAP	Promotion of cell proliferation and migration	[79]
RB	has_circ_0000034	miR-361-3p/STX17	Promotion of cell growth	[80,81]
RB	circMKLN1	miR-425-5p/PDCD4	Inhibition of tumor progression	[82]
RB	circ-FAM158A	miR-138-5p/SLC7A5	Regulation of cell growth and migration	[83]
RB	circ_0084811	miR-18a-5p/miR-18b-5p/E2F5	Promotion of proliferation and inhibition of apoptosis	[84]

MB, medulloblastoma; EP, ependymoma; NB, neuroblastoma; OS, osteosarcoma; ERMS, embryonal rhabdomyosarcoma; ARMS, alveolar rhabdomyosarcoma; WT, Wilms’ tumor; HB, hepatoblastoma; RB, retinoblastoma.

## 3. Brief View on circRNAs in Blood Cancers

Although we focused our attention on the latest scientific evidence regarding the identification and possible role of circRNAs in different solid pediatric tumors, a brief consideration of their involvement in blood cancers must be made. Indeed, circRNAs could be applied as new clinical biomarkers for blood cancers due to their close connections with multiple biological functions in the disease, as well as their elevated levels of stability and abundance in bone marrow and body fluids [85]. Several circRNAs have been used as diagnostic and prognostic biomarkers in blood malignancies, such as multiple myeloma (MM), acute lymphocytic leukemia (ALL), and juvenile myelomonocytic leukemia (JMML). For example, in MM, circRNAs involved in cell proliferation and tumor progression (such as circ-SMARCA5, hsa_circ_0007841, hsa_circ_0005273, hsa_circ_0007146, hsa_circ_0001947, hsa_circ_0001910, circ-CDYL, circ-MYBL2, circ-ITCH, hsa_circ_0087776, circMYC, circ-G042080, circ-ATP10A) [86] or in chemoresistance (such as circRNA_101237, circ_0007841, circITCH, circ-CCT3, ciRS-7, circPVT1) were identified [87]. In ALL, circPVT1 was proposed to promote ALL leukemogenesis [88], while circPVT1, circHIPK3, and circPAX5 were proposed to interfere with B cell maturation and promote disease progression [89]. In 2021, Liu et al. identified circRNF220, which was specifically abundant and accumulated in the peripheral blood and bone marrow of pediatric patients with AML. Interestingly, they suggested the use of this circRNA as a prognostic biomarker because its elevated expression correlated with relapse. Moreover, they found that circRNF220 knockdown specifically inhibited proliferation and promoted apoptosis in AML cell lines and primary cells [90]. In JMML, CircMCTP, circLYN, and circAFF2 were highly upregulated, suggesting their involvement in this tumor’s pathogenesis [91]. As in solid pediatric tumors, the search for circRNAs in blood malignancies is still in its infancy and is an important resource for the identification of biomarkers on which the spotlight is turned on.

## 4. Bioinformatic Tools and Resources for circRNAs

Identification of circRNAs in RNA sequencing data remains a major challenge, especially for their detection within pooled heterogeneous RNAs. In fact, circRNAs generally lack poly(A) tails [19], even though some A-rich stretches can still be found within their sequences. Random priming can be used in place of oligo(dT) priming to enrich circRNA libraries. Moreover, despite circRNAs are retained in rRNA-depleted libraries, they could require the use of RNase R to digest linear RNA. In addition, adaptor ligation could represent an artifact in circRNA enrichment as two different cDNAs may be ligated in a noncanonical order during the step of adaptor ligation. Another problem is template switching that is promoted in case of long homologous sequences, such as those found in the genes coding for multiple isoforms that share identical constitutive exons [92,93]. All of these experimental artifacts should be taken into account by the algorithms that are used to identify circRNAs. To date, different algorithms have been developed to resolve these issues [94], each one with its own methodologies. The most common algorithms are listed in Table 2 [93]. These are used for single-end or paired-end data, with the latter being more sensitive, especially when dealing with high read coverage [95]. Reads that are not contiguously aligned to the reference genome and that align to back-splice junctions are further processed by distinct algorithms in order to identify and count circRNAs. A list of bioinformatic resources was developed to allow not only the discovery, but also the study of the mechanism of function of circRNAs. These tools comprise both those for circRNA identification and quantification and resources for circRNA annotation. Indeed, there are several databases that collect circRNA information related to many aspects of this RNA’s biology, such as interaction with other biological entities as well as expression data across cells and tissues and tools for experiment design. Such resources were comprehensively reviewed by Chen et al. [96].

## 5. Conclusions

In the past few years, the advancement of such high-throughput technologies as sequencing and omics techniques led to the identification of circRNAs. These conserved endogenous RNAs have an extensive distribution, even though they present tissue-specific expression, cell type specificity, and multiple functions. Intriguingly, dysregulated expression of circRNAs has been identified in various cancers. Even though little is known about their function and molecular mechanisms during cancer initiation, progression, and metastasis, circRNAs have a great potential as cancer biomarkers. Indeed, the emerging involvement of circRNA deregulation in cancer pathogenesis has opened promising opportunities for their clinical application in tumor diagnosis, outcome prediction, and therapy. Even though circRNAs arouse considerable interest and are under the magnifying glass, their characterization and study are still in their infancy, and many questions still remain unanswered. It is highly unlikely that any single circRNA, despite being highly sensitive and specific, exists in all cancer types, and one specific circRNA is usually insufficient to predict or monitor cancer; therefore, integrating a panel of cancer-associated circRNAs as biomarkers or signatures might be a reasonable method in future clinical practice, in particular in pediatric cancer. Indeed, the current challenges in the field of childhood cancer diseases include identification of novel biomarkers that may allow a more accurate risk stratification. These circular molecules, specific and extremely stable, implicated in the regulation of physiological processes and deregulated in several pathologies, such as cancer, serve as stable clinical biomarkers of disease and also provide new potential therapeutic targets in the pediatric setting.

## Figures and Tables

**Table 2:** Tools for circRNA identification and annotation.

Algorithm	Aligner	Reference
find_circ	Bowtie2	[6]
MapSplice	Bowtie	[97]
CIRCexplorer	TopHat	[16]
circRNA_finder	STAR	[98]
CIRI	BWA	[99]
Salzman 2012	Bowtie	[100]
Salzman 2013	Bowtie2	[101]
CircRNAseq	Bowtie	[15]
Segemehl	Segemehl	[102]
Guo 2014	Bowtie	[103]
KNIFE	Bowtie2	[104]
DCC	STAR	[105]

## Data Availability

Not applicable.
